# The effect of missing levels of nesting in multilevel analysis

**DOI:** 10.5808/gi.22052

**Published:** 2022-09-30

**Authors:** Seho Park, Yujin Chung

**Affiliations:** 1 Department of Biostatistics and Health Data Science, Indiana University School of Medicine, Indianapolis, IN 46202, USA; 2 Department of Applied Statistics, Kyonggi University, Suwon 16227, Korea

**Keywords:** hierarchical structure data, missing levels of nesting, multilevel model

## Abstract

Multilevel analysis is an appropriate and powerful tool for analyzing hierarchical structure data widely applied from public health to genomic data. In practice, however, we may lose the information on multiple nesting levels in the multilevel analysis since data may fail to capture all levels of hierarchy, or the top or intermediate levels of hierarchy are ignored in the analysis. In this study, we consider a multilevel linear mixed effect model (LMM) with single imputation that can involve all data hierarchy levels in the presence of missing top or intermediate-level clusters. We evaluate and compare the performance of a multilevel LMM with single imputation with other models ignoring the data hierarchy or missing intermediate-level clusters. To this end, we applied a multilevel LMM with single imputation and other models to hierarchically structured cohort data with some intermediate levels missing and to simulated data with various cluster sizes and missing rates of intermediate-level clusters. A thorough simulation study demonstrated that an LMM with single imputation estimates fixed coefficients and variance components of a multilevel model more accurately than other models ignoring data hierarchy or missing clusters in terms of mean squared error and coverage probability. In particular, when models ignoring data hierarchy or missing clusters were applied, the variance components of random effects were overestimated. We observed similar results from the analysis of hierarchically structured cohort data.

## Introduction

A multilevel model has gained popularity as a practical and essential analysis tool in various fields such as epidemiological research, public health research, and social or educational research [1–6]. A specific study design involving multiple levels or nested structures results in a hierarchical structure and accompanies the application of multilevel modeling [1]. Multilevel data are often obtained as an incomplete dataset with missing components at any level of the data hierarchy. For example, in practice, a nesting of some hospitals and practices, which are also nested within larger health systems, can be ignored or unobserved in the health care study [7].

In practice, researchers in the field ignore a hierarchy within the data and prefer an ordinary least square regression model (OLS) that treats all observations as if they are measured at the same level [8–12]. Alternatively, a two-level model is also commonly adopted by ignoring missing intermediate levels and accounting for only the top and bottom levels of hierarchy in multilevel data. However, the misleading hierarchy by ignoring intermediate or top levels may potentially impact the parameter estimation in multilevel analysis and has gained attention among researchers in recent research [13–17].

To handle the missing issues, several studies proposed imputation methods to fill in the missing and create a complete dataset [15–17]. However, imputation methods add more uncertainty to the parameter estimations and especially increase the complexity when the imputation is intertwined with the hierarchical structure of the data [15–18]. In particular, it is difficult to handle the missing when a cluster is entirely missing or the cluster size is very small, and especially the missing values are in the explanatory variables. Alternatively, a single imputation can be considered. It is because it handles the practical issues in multilevel imputation, is simple to adapt, and preserves the design hierarchy of the multilevel data. In this study, we consider a multilevel linear mixed effect model (LMM) with a single imputation that was introduced in Sanders' study [19] for handling missing intermediate or top clusters in multilevel data. An LMM has been widely accepted to model the hierarchical structure in multilevel data and can account for the correlation among units nested within clusters. The method uses a single imputation that replaces the missing clusters by its nesting lower-level unit’s measurements and considers as if each of the nesting lower-level unit’s measurements is a single observation for missing clusters. As a result, a missing intermediate-level cluster is filled in containing only a singleton, resulting in a complete dataset [19]. However, to our best knowledge, no previous studies have examined and investigated the performance of an LMM with a single imputation and directly compared it with other models that ignore missing levels of nesting.

This study aims to evaluate the performance of a model considering all hierarchies, such as an LMM with single imputation, in three-level data and demonstrate that it outperforms other models that do not match the hierarchy of multilevel data. To do so, we compare three models in three-level hierarchical data with missing intermediate-level clusters. The first model is an OLS regression model, which is a single-level model ignoring any hierarchy in the data and treats all observations measured at the same level. Secondly, we consider a two-level LMM considering only level-1 and level-3 and discarding missing intermediate levels. The third model is a three-level LMM with a single imputation that involves all levels of the data hierarchy, so we can assume that the multilevel model matches the design hierarchy in the data by filling in a missing level-2 unit.

In this study, we apply and compare the three models to the Childhood to Adolescence Transition Study (CATS), a motivating case study of this work, in a three-level structure: school (level-3 unit), individual (level-2 unit), and repeated measures observed per individual (level-1 unit) with missing level-2 and level-3 units [20,21]. We also conduct a thorough simulation study by comparing the three models to examine the impact of ignorance of missing intermediate level on parameter estimation of three-level data analysis when the missing rate and the cluster sizes of level-2 and level-3 clusters vary.

## Methods

### Three-level linear mixed effect model

In the context of multilevel data analysis, a LMM is considered as an analysis model that accounts for correlation due to the hierarchical structure of the data. To establish background information on the analysis model for hierarchical structure data, especially for a three-level data structure, we develop a general notation and introduce a brief overview of a three-level model.

Let *i* be the index of the level-3 unit with size *L* (*i*=1,···,*L*), *j* be the index of the level-2 unit with size *M_i_* (*j*=1,…,*M_i_*), and *k* be the index of the level-1 unit with size *N_ij_* (*k*=1,…,*N_ij_*). We consider a three-level model, where a level-1 unit is nested within a level-2 unit that is also nested within a level-3 unit. Let *y_ijk_* be the response variable for level-1 unit *k* in the level-2 unit *j* and level-3 unit *i*, *x_ijk_* be the associated covariate variable observed at level-1, *x_ij_* be the associated covariate variable at level-2, and *x_i_* be the associated covariate at level-3. Then a general three-level linear mixed effect model is:


(1) $$\begin{array}{ll} y_{ijk}\; & =\beta_{0ij}+\beta_{1ij}x_{ijk}+e_{ijk}\\ \beta_{0ij}\; & =\alpha_{0i}+\alpha_{01}x_{ij}+v_{ij}\\ \beta_{1ij}\; & =\alpha_{1i}+\alpha_{11}x_{ij}+{v^{\prime }}_{ij}\\ \alpha_{0i}\; & =\gamma_{00}+\gamma_{01}x_{i}+u_{i}\\ \alpha_{1i}\; & =\gamma_{10}+\gamma_{11}x_{i}+{u^{\prime }}_{i}, \end{array}$$

where $e_{ijk}\sim N(0,\sigma_{e}^{2})$ are residuals at level-1 that are independent and identically distributed (*iid*) and capture within-cluster residual variations, ***v****_ij_*=(*v_ij_*,*v*′*_ij_*) are *iid* random effects at level-2 and captures between-cluster intercept differences at level-2, ***u****_i_*=(*u_i_*,*u*′*_i_*) are *iid* random effects at level-3 and captures between-cluster intercept differences at level-3.

In this study, we assume that only the regression intercept varies across clusters and hence restrict our attention to a three-level random effect model with a random intercept for each level. It is the simplest type of linear mixed model for multilevel study, although we note that an extended model including random slope can be considered. We revisit this in the discussion. Considering a random effect model with random intercepts only, *α*_11_=*v*′*_ij_*=*γ*_11_=*u*′*_i_*=0. Therefore, the model in Eq. (1) can be rewritten as


(2) $$y_{ijk}=\beta_{0}+\beta_{1}x_{i}+\beta_{2}x_{ij}+\beta_{3}x_{ijk}+u_{i}+v_{ij}+e_{ijk},$$

where *β*_0_(=*γ*_00_) is the intercept, and *β*_1_(=*γ*_01_),*β*_2_(=*α*_01_), and *β*_3_(=*γ*_10_) are the slope coefficients for the fixed effects at level-3, level-2, and level-1, respectively. Considering random intercepts only in the model, we assume that level-3 and level-2 residuals, *u_i_* and *v_ij_*, respectively, are multivariate normal with zero means and variances of $\sigma_{u}^{2}$ and $\sigma_{v}^{2}$, respectively, and the within-cluster residuals are normally distributed with mean zero and constant variance $\sigma_{e}^{2}$:


$$u_{i}\sim N(0,\sigma_{u}^{2}),\;v_{ij}\sim N(0,\sigma_{v}^{2}),\;e_{ijk}\sim N(0,\sigma_{e}^{2}).$$

The covariance structure of the response vector ***y***=(*y*_111_,*y*_112_,…,*y*_*L*_,*M*_*L*_,*N*_*LM*_) is a block-diagonal covariance matrix given by


$$Cov\left(y\right)=\left[\begin{array}{ccc} G_{1}+\sigma_{u}^{2}J_{\left(n_{1}\right)} & & \\ & \ddots & \\ & & G_{L}++\sigma_{u}^{2}J_{\left(n_{L}\right)} \end{array}\right]$$

with an *i*th block-diagonal matrix of $G_{i}=\sigma_{e}^{2}I_{\left(n_{i}\right)}+\sigma_{v}^{2}J_{\left(n_{i}\right)}$, where ***I***_(*n*_*i*__ is an *n_i_*×*n_i_* identity matrix, ***J***_(_*_n_*_*_i_*)_ is an *n_i_*×*n_i_* matrix of 1s, and and *n_i_*=*M_i_*×*N_iM_*_*_i_*_ is a size of a block-diagonal matrix associates with *i*th level-3 cluster. The off-diagonal elements are zero.

### Analysis of incomplete multilevel data using single imputation

To handle the missing level-2 units in a three-level hierarchical data, we adopt a single imputation method introduced in Sanders’ study [19] that replaces the missing clusters by its nesting lower-level unit’s measurements and considers the imputed level-2 unit as a cluster containing a single observation. Specifically, it constructs imaginary level-2 clusters for level-1 measurements whose level-2 clusters were missing entirely. As a result, the single imputation method adds level-2 clusters as many as the number of level-1 units directly under the missing level-2 clusters. By applying the method, we can have a complete three-level dataset without any missing clusters at level-2 units, allowing us to apply a three-level model. The single imputation method allows us to preserve a hierarchical data structure in multilevel data analysis.

The motivating case study CATS has a three-level structure with the components of school, individual, and its repeated measures per individual, and some measurements at the individual level (level-2; academic numeracy score at baseline) were partially not observed and recorded as missing in the dataset. There were 22.4% missing academic numeracy scores at baseline at level-2, and they were filled in by single imputation that impute a corresponding academic numeracy score measured at level-1 to handle the components with missingness. In other words, we treated each level-1 unit within a missing level-2 unit is nested in a level-2 unit that only contains the level-1 unit itself [19].

### Childhood to Adolescence Transition Study

Motivated by the case study of CATS, we focus on an analysis of the simulated data mimicking the CATS data [21]. The CATS is a longitudinal cohort study collected through multiple waves, and it collects mental health to investigate the effect of early depressive symptoms on academic outcomes in children from puberty through adolescence. More details about the data collection and study protocol can be found in Mundy et al. [20].

The simulated data mimicking the CATS have a three-level hierarchical structure. Individuals are nested within schools, and repeated measures within individuals were collected. The data consist of demographic, educational, and social outcomes as well as mental health outcomes for the CATS study: depressive symptoms, National Assessment Programme – Literacy and Numeracy results (NAPLAN) academic numeracy score, socio-economic status, age, and sex are collected for the study [21].

The NAPLAN numeracy score is an outcome of interest and is observed at level 2 at wave 1 (potential baseline) and at level 1 in the following waves with missing cases. As we are interested in a case where missingness happens at level-2 only, we discard cases where the NAPLAN numeracy score has missingness at level-1, and the size of data that we used is N = 2,592. The data are unbalanced as the size of level-2 differs, and the number of level-1 units within a level-2 unit varies across individual. The dataset has 163 schools; 54 of 163 schools have a single individual with a single measurement per individual, and the other 109 schools have multiple individuals with a different number of repeated measures. There were 1,142 individuals in the data, and 256 of 1,142 (22.4%) NAPLAN numeracy scores at the individual level are missing.

### Simulation data

We conducted a simulation study to evaluate the performance of a three-level LMM with single imputation. In the simulation, we considered complete three-level data sets and incomplete data with various missing rates of level-2 clusters (Table 1) and compared the performance of the three-level LMM with single imputation with those of a single-level model and 2-level LMM.

We generated complete data sets without missing subjected to the model in Eq. (2), where we assumed the fixed coefficients of *β*_0_=0.5, *β*_1_=*β*_2_=*β*_3_=0.3, the random effects at level-2 and level-3 of $u_{i}\sim N(0,\sigma_{u}^{2})$ and $v_{ij}\sim N(0,\sigma_{v}^{2})$ with *σ_u_*=1.4 and *σ_v_*=1, respectively, and $e_{ijk}\sim N(0,\sigma_{e}^{2})$ with *σ_e_*=1.4 for *i*=1,…,*L*, *j*=0,1,…,M and *k*=1,…,*N_ij_*. We considered various cluster sizes at each level (Table 1). The number of level-3 clusters varied as *L*=30,50, and 100. For each level-3 cluster, we assumed *M*=10,30 clusters. The number of level-1 clusters within each level-2 cluster was randomly determined as *N_ij_*~*Unif*[15,40]. For all cases, the covariates were generated as *x_i_*~*N*(0,1), *x_ij_*~exp(1)+1, and *x_ijk_*~*N*(1,1.2^2^). Intraclass correlations were set to 0.398 and 0.602 for level-3 and level-2, respectively. For each case, we generated 500 replicates.

To evaluate the performance of the three-level LMM in the presence of missingness at the level-2 units, we generated missingness from each of the complete 3-level data sets (Table 1). We set the complete data to be missing in *M* level-2 clusters and its related variables such as *x_ij_*. The missing data rate in level-2 clusters was set to 10, 25, 50, and 75% of *M* level-2 clusters.

## Results

### Childhood to Adolescence Transition Study

We use three analysis models to determine the effect of ignorance of hidden levels in the hierarchically structured CATS data: (1) a single-level model (ordinary least square regression) that completely ignores the structure of the hierarchy and treats the data collected at level-1 only (M1), (2) a two-level LMM with a random effect for school (level-3) only and ignoring an individual (level-2) random effect (M2), and (3) a three-level LMM with single imputation considering all levels of hierarchy in the CATS study units (M3). The LMMs we fit for M2 and M3 only have random intercepts, and random slopes are not considered in the data analysis. Tables 2 and 3 report estimates of the fixed and random effects, respectively, across the three models.

Table 2 presents estimated coefficients of fixed effects in each model, and Table 3 presents the estimated variance components of random effects and residuals. Note that M2 has one random effect at school and M3 has two random effects at school and individual.

Based on a significance level of 5%, depressive symptoms appeared to be significantly meaningful in supporting the effect of mental health on academic outcomes in children from puberty through adolescence across the three models (p < 0.001). Age and NAPLAN numeracy scores observed at baseline were meaningful covariates for explaining the relationship with academic outcome in addition to the mental health outcome. That is, when children are young, have fewer depressive symptoms, and higher NAPLAN numeracy scores at baseline, academic outcomes are likely to be higher. Noticeably, the estimated coefficients of the important covariates do not differ much across the three analysis models.

Table 3 presents the estimated variance components of random effects and residuals in the multilevel data across the three analysis models with different hierarchy levels. The estimated residual variance quantifies the variation within repeated measures and decreases when random effects are included in the analysis model (M2 and M3). The estimated variance components of the random effects explain variations across schools and individuals that were not explained by M1, and the sum of the estimated variance components in M3 is larger than in M2. This implies that M3 captures more unexplained variation in the hierarchical structure data, especially the variation among individuals, which cannot be ignorable. Hence, the analysis without accounting for the variance among the unobserved level of nesting units in data analysis resulted in a larger estimation of the residual variance as shown in Table 3. It might lead to an inaccurate estimation of the total variation in the multilevel data.

### Simulation data

Considering 30 scenarios (Table 1), we applied a single-level model (M1) and 2-level LMM (M2) and 3-level LMM with single imputation (M3). M1 and M2 estimate four fixed coefficients, and the standard deviation $\sigma_{e}^{2}$ of the error term but does not estimate some of the variances of the random effects, while M3 estimates all parameters described in Eq. (2). Since M1 ignores the hierarchical structure of the data, it does not contain the random effect terms of *u_i_* and *v_ij_*. M2 considers level-1 and level-3 clusters by assuming that level-1 units are directly connected to level-3 clusters. Therefore, M2 does not include the random effect term of *v_ij_*. In summary, M3 only estimates $\sigma_{v}^{2}$, and M2 and M3 models estimates $\sigma_{u}^{2}$. The estimates of all fixed parameters were compared to the true values of the parameters used to simulate the data. In particular, we calculated the mean squared error (MSE) across 500 replications, and the coverage probability was estimated by the proportion of replications with 95% confidence intervals containing the true value.

We expected that M3 performed well in the presence of unobserved level-2 clusters, whereas M2 would poorly estimate the coefficient β_2_ of the level-2 associated covariate with missing clusters since M2 ignored the level-2 clusters and partially incorporated the date structure of level-1 and level-3 clusters. Since the M1 did not consider the hierarchical data structure, we expected that it poorly estimated *β*_1_ and β_2_.

**Fig. 1:**
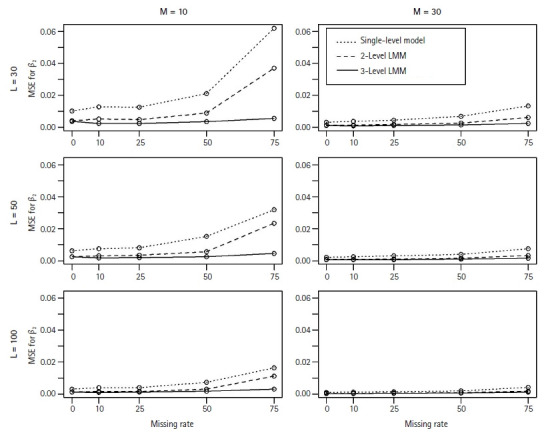
The MSEs for β_2_, the coefficient of the level-2 associated covariate, when estimated by the single-level model (···), 2-level LMM (---), or 3-level LMM (─). For each plot of L (=30, 50, 100) level-3 clusters and M (=10, 30) level-2 clusters, each point represents the MSE across 500 simulated data sets over a range of missing rates in level-2 clusters. MSE, mean squared error; LMM, linear mixed effect model.

**Fig. 2:**
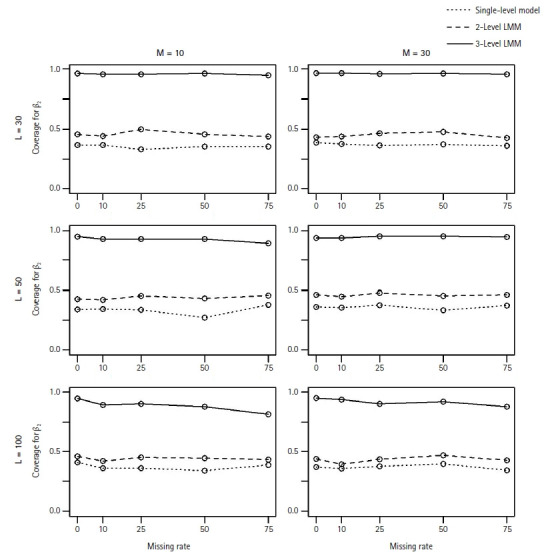
The coverage probability for β_2_, the coefficient of the 2-level associated covariate, when estimated by the single-level model (···), 2-level LMM (---), or 3-level LMM (─). For each plot of L (=30, 50, 100) level-3 clusters and M (=10, 30) level-2 clusters, each point represents the coverage probability across 500 simulated data sets over a range of missing rates in level-2 clusters. LMM, linear mixed effect model.

In general, the M3 performed well and better estimated the fixed coefficients in Eq. (2) than the M1 and M2 models. As expected, in particular, we observed that the M1 and M2 models poorly estimated the coefficient β_2_ of the level-2 associated covariate with missing clusters. Figs. 1 and 2 show the MSEs and the coverage probabilities for β_2_, respectively, and compared those values by the three models. The MSEs by the M3 ranged from 0.00039 to 0.00557, while those by the M1 and M2 were 0.0011–0.0639 and 0.0004–0.037, respectively (Fig. 1). The coverage probabilities for β_2_ by the M3 were approximately 0.814–0.964 (Fig. 2). The M1 and M2 models did poorly estimate β_2_ and their coverage probabilities low as 0.27–0.406 and 0.392–0.498, respectively (Fig. 2). The MSEs for β_2_ seemed to increase with the missing rate (Fig. 1); however, the coverage probabilities did not change as the missing rate increased.

We observed MSEs for *β*_0_, β_1,_ and β_3_ similar to Fig. 1. The differences in MSEs among the three models were small, but M3 performed the best overall. Moreover, MSEs tended to increase with missing rates. Similar to Fig. 2, the coverage probabilities for *β*_0_ and β_1_ were around 0.95 from the M3, while we observed small coverage probabilities from M1 (e.g., 0.06–0.302 for β_1_) and comparable coverage probabilities from M2 (e.g., 0.926–0.96 for β_1_). The three models yielded similar coverage probabilities for *β*_3_.

The three models contain different variance components. M3 can estimate the three standard deviations, *σ_u_*, *σ_v_* and *σ_e_*. However, M2 does not estimate the variance $\sigma_{v}^{2}$ among level-2 clusters, and M1 can estimate the variance $\sigma_{e}^{2}$ among individuals but not *σ_u_* and *σ_v_*. We expected that the M1 and M2 models could overestimate variance components by failing to explain the 3-level data structure.

**Fig. 3:**
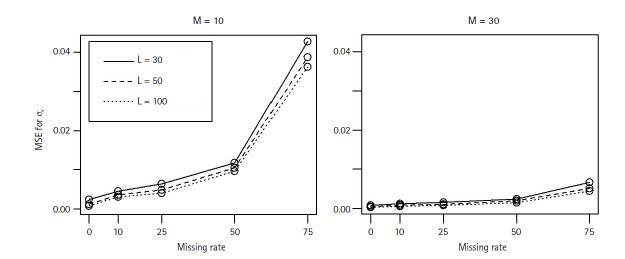
The MSEs for σ_v_ from the 3-level LMM (M3) when the level-3 cluster size changes as L = 30 (···), L = 50 (---), or L = 100 (─). For each case of M = 10 or 30 level-2 cluster sizes, 500 datasets were simulated over a range of missing rates in level-2 clusters. MSE, mean squared error; LMM, linear mixed effect model.

**Fig. 4:**
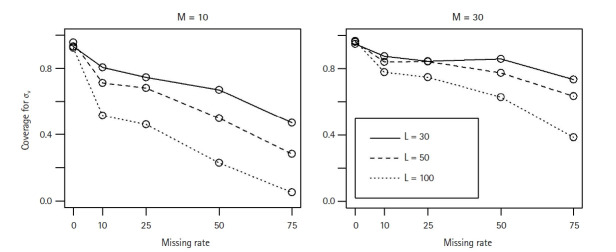
The coverage probability for σ_v_ from the 3-level LMM (M3) when the level-3 cluster size changes as L = 30 (···), L = 50 (---), or L = 100 (─). For each case of M = 10 or 30 level-2 cluster sizes, 500 datasets were simulated over a range of missing rates in level-2 clusters. LMM, linear mixed effect model.

Figs. 3 and 4 show that the MSEs and coverage probabilities for *σ_v_* from M3 worsened as the missing rate increased for each case of L and M values. At the same missing rate, the larger value of M, the better the coverage. However, more level-3 clusters did not improve MSE and coverage probability. This is because each level-3 cluster contains unobserved level-2 clusters, and hence the number of unobserved level-2 clusters increases as L increases.

**Fig. 5:**
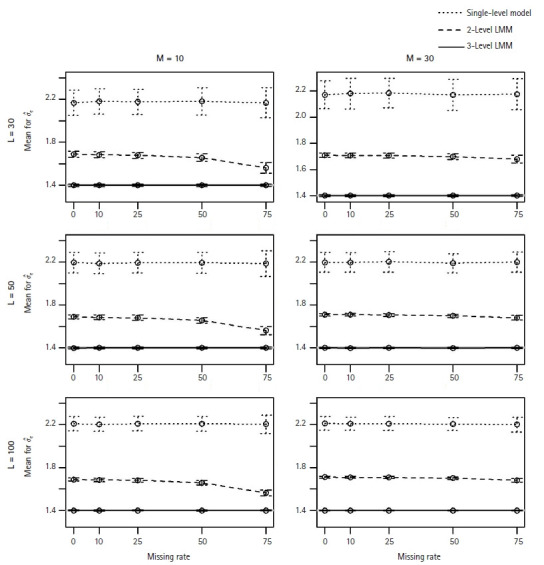
The average estimates for σ*_e_* when estimated by the single-level model (···), 2-level LMM (---), or 3-level LMM (─). For each plot of L (=30, 50, 100) level-3 clusters and M (=10, 30) level-2 clusters, each point represents the mean of estimates across 500 replicates, and vertical lines indicate the standard errors. The true value σ*_e_* is 1.4. LMM, linear mixed effect model.

We observed that the estimates for *σ_u_* and *σ_e_* from M1 were unbiased with small MSEs and their coverage probabilities were close to the nominal level of 95% (0.918–0.96 for *σ_u_*; 0.936–0.974 for *σ_e_*). However, the coverage probabilities of M1 and M2 models were very low (e.g., 0–0.344 for *σ_u_* and 0–0.006 for *σ_e_* from the 2-level model), and *σ_u_* and *σe* were overestimated by M1 and M2 models (see Fig. 5 for the average estimates for *σe*).

## Discussion

In this study, we investigated the effect of missing intermediate level of the hierarchical structure data with varying missing rates, as well as the consequence of ignorance of the level of nesting on the parameter estimation of multilevel analysis. Since missing level-2 units are imputed by the nested level-1 unit’s measurement, the intermediate-level unit has a single observation per unit, and the data can be considered sparse. We compared three models with different levels of hierarchy across varying missing rates at the intermediate-level and various cluster sizes in the top- and intermediate-level clusters. We observed that the three-level LMM with single imputation showed a better performance compared to the other two models, which ignore higher levels of nesting in terms of MSE and coverage. Moreover, lower-level variance components were overestimated, indicating that variance components are affected by ignorance of the intermediate level.

We considered a random effect model for the 2-level and 3-level linear mixed effect models in the simulation study and data application for brevity, and it can be expanded to consider a random slope model and/or interaction terms. However, it requires caution to generalize the model analysis to be more complex because we have a large number of intermediate-level clusters with a single observation, and such sparse clusters might lead to biased estimates in random slope estimation [22]. Also, we have incomplete variables for intermediate-level units. An imputation requires careful caution when incomplete explanatory variables with a random slope and/or interaction terms exist in the model because they might lead to invalid estimates [10].

Furthermore, we used a three-level LMM with single imputation that replaces the missing by the nesting lower-level unit’s measurements and considering that a single observation is measured per intermediate-level unit. As a future study, other imputation methods for multilevel data can be considered and compared their performances with an approach studied in this work. In practice, it is common that level-1 and level-2 units are both missing, and in such case, the imputation method, such as a multiple imputation handling multiple level’s missingness, should be considered.

## Figures and Tables

**Table 1:** Simulation schemes to generate complete 3-level data and incomplete data with missing in level-2 units

**Complete 3-level data sets**
–Given level-3 cluster size L (=30, 50, 100) and level-2 cluster size M (=10, 30), –Generate level-1 cluster size *N_ij_*~*Unif*[15,40]. –Given *N_ij_*, generate a data set (*y_ijk_*,*x_i_*,*x_ij_*,*x_ijk_*) from the model in Eq. (2).
**Incomplete 3-level data sets with missing in the level-2 units**
–Given each complete data set and missing rate *p* (=10, 25, 50, 75%). –Randomly select and discard × *px_ij_*’s from the complete data set. –Then an incomplete data set consists of *M × p* observations of (*y_ijk_*,*x_i_*,*x_ijk_*) and *M ×* (1−*p*) observations of (*y_ijk_*,*x_i_*,*x_ij_*,*x_ijk_*).

We note that complete 3-level data sets can be considered as the case of a 0% missing rate. In total, we considered 30 simulation scenarios (three level-3 cluster sizes × two level-2 cluster sizes × five missing rates with a range of 0%, 10%, 25%, 50%, and 75%).

**Table 2:** Estimates of coefficients for CATS data inferred by the three models: single-level model (M1), two-level LMM (M2), and three-level LMM (M3)

	M1			M2			M3		
	Coef	SE	p-value	Coef	SE	p-value	Coef	SE	p-value
(Intercept)	2.789	0.225	<0.001	2.744	0.212	<0.001	2.748	0.234	<0.001
Depressive symptom^a^	−0.043	0.013	<0.001	−0.047	0.012	<0.001	−0.048	0.010	<0.001
Age^a^	−0.173	0.023	<0.001	−0.172	0.022	<0.001	−0.173	0.025	<0.001
Sex^a^	0.037	0.041	0.371	0.018	0.038	0.633	0.021	0.044	0.901
SES^a^	0.019	0.021	0.360	0.011	0.019	0.579	0.006	0.022	0.764
NAPLAN at baseline^b^	0.677	0.021	<0.001	0.675	0.019	<0.001	0.676	0.021	<0.001

CATS, Childhood to Adolescence Transition Study; SE, standard error; SES, socio-economic status; NAPLAN, National Assessment Programme – Literacy and Numeracy results.

a Covariate at level-1.

b Covariate at level-2.

**Table 3:** Estimated variance components of random effects and residuals for CATS data inferred by the three models: single-level model (M1), two-level LMM (M2), and three-level LMM (M3)

Variance component	M1	M2	M3
Level 3: school	-	0.216	0.137
Level 2: individual	-	-	0.325
Residual	1.002	0.786	0.426

CATS, Childhood to Adolescence Transition Study; LMM, linear mixed effect model.
